# Sex disparities in health of older adults in India: assessing the morbidity-mortality paradox through disability-free life expectancy

**DOI:** 10.1186/s41118-025-00247-2

**Published:** 2025-05-13

**Authors:** Sadanand Karun, Lotus McDougal, Abhishek Singh

**Affiliations:** 1 International Institute for Population Sciences, Mumbai, India; 2 Center On Gender Equity and Health, University of California San Diego, La Jolla, USA; 3 Department of Public Health and Mortality Studies, International Institute for Population Sciences, Mumbai, India

**Keywords:** Life expectancy, Morbidity-mortality paradox, Healthy life expectancy, Disability-free life expectancy, Gender disparity, Stepwise replacement decomposition, India

## Abstract

**Background:**

Older adults face substantial sex gaps in health. In many contexts, females live longer than males, but their time spent with disability is also higher. Our study assesses (i) the sex gap (female–male) in health through life expectancy and healthy life expectancy and (ii) the morbidity-mortality paradox among older adults aged 60 and above in India and its states.

**Methods:**

We utilized data on age-specific death rates obtained from the Sample Registration System and age-specific disability prevalence from the Longitudinal Ageing Survey (LASI) in India. We estimated abridged life tables between age groups 60–64 to 85 + using Greville’s method. We then combined the disability data obtained from LASI with the constructed life tables using Sullivan’s method to estimate disability-free life expectancy (DFLE) and life expectancy with disability (DLE). Finally, we decomposed the sex gap in DFLE and DLE into mortality and disability components using a stepwise replacement decomposition method.

**Results:**

At the national level, life expectancy at age 60 for males were 17.4 years and for females 19.2 years, indicating a female mortality advantage of 1.8 years. At the state level, the sex gap ranged between 5.1 years in Jammu & Kashmir and -1.1 years in Jharkhand. The disability prevalence was higher among females compared to males at the national level and in all states. The decomposition result indicates that 98% of the mortality advantage of females at the national level was spent in disability. The disability disadvantage of females over their mortality advantage was highest in Uttar Pradesh; 93% of additional years of life were spent with disability. The disability disadvantage of females over their mortality advantage was lowest in Rajasthan where only 9% of additional years were spent in disability. Stepwise replacement decomposition of the sex gap in DFLE by age groups shows that as age increases, the contribution of mortality effects decreases, whereas disability effects increase.

**Conclusions:**

We find evidence of a morbidity-mortality paradox in India nationally and sub-nationally. As the sex gap in health and its implications vary across the states of India, policies to address these inequities must also vary across the states.

**Supplementary Information:**

The online version contains supplementary material available at 10.1186/s41118-025-00247-2.

## Introduction

Significant improvement in mortality reduction has been made worldwide, resulting in longer life expectancies and aging populations. Globally, life expectancy at birth for females have been estimated at 74.8 years in 2021, which is 5.8 years longer than males (Ferrari et al., 2024). In addition, studies indicate that females live longer than males across countries (Ferrari et. al., 2024; Wang et al., 2020). However, despite living longer, females experience more non-fatal health conditions such as low back pain, musculoskeletal-mental-anxiety disorders, and disabilities compared to males (Patwardhan et al., 2024). This phenomenon of female living longer than male (mortality advantage) but simultaneously being more prone to having worse health (disability disadvantage) than male is widely known by several names, including the ‘male-female health-survival paradox’, the ‘gender and health paradox’, and the ‘morbidity-mortality paradox’ (Gorman et al., 2006; Kulminski et al., 2008; Lego et al., 2021; Rieker & Bird, 2005).

In the Indian context, significant progress has been made in terms of mortality reduction reflected by increasing life expectancies. For instance, life expectancy (LE) at birth has increased by 8.6 years for male and 10.6 years for female between 1990 and 2016 (Dandona et al., 2017). During the early 1970s, LE at birth among males in India was one year more than females; this ratio has since reversed, with females now outliving males. India’s *Sample Registration System (SRS) Based Abridged Life Table Report 2020* shows females’ survival at birth surpassing males’ survival by 1.5 years in the year 2000. The sex gap in LE at birth increased further and reached 2.8 years (LE at birth for females: 71.4 years; LE at birth for males: 68.6) in the year 2020 (ORGI, 2020).

Despite living longer than males, females suffer more disease and disability than males, an inequity which is more pronounced at age 60 and above. Studies focused on assessing the health status of Indian older adults establish that females have significantly poorer cognitive health, weak handgrip strength, and poor self-rated health (Akhtar et al., 2023; Oksuzyan et al., 2018; Singh et al., 2018). Moreover, older females tend to have higher levels of health issues such as depression (Paul et al., 2023), noncommunicable diseases (Chauhan et al., 2022; Sharma et al., 2023), and functional limitations (Malik, 2022; Sengupta & Agree, 2002) compared to their male counterparts.

This morbidity-mortality paradox raises the question of whether females’ mortality advantage is really translating into a corresponding advantage in health or instead is forcing them to spend more time in poor health and resultant diminished quality of life. The concept of healthy LE was developed to answer this question by combining mortality and morbidity components. Healthy LE is a summary measure of population health used to understand the differentials in health across population sub-groups by combining health (~disability or morbidity) with mortality rates. Disability-free life expectancy (DFLE) and disability life expectancy (DLE) are two crucial measures of healthy LE, which are derived by disaggregating total LE. By definition, DFLE is the average number of years a person could expect to live in good health without any activity limitation at a given age in a geographical area, given the mortality rates and disability prevalence levels at that age and area. In contrast, DLE is the average number of years one can expect to live in poor health at a certain age x, given the mortality rates and disability prevalence at that age (Lu et al., 2019; Murray, 2002; Sanders, 1964; Shabnam & Saikia, 2024; Sullivan, 1971).

Several studies have been conducted worldwide using the concept of healthy LE in general and DFLE in particular (Andrade et al., 2011; Crimmins et al., 2016; Nepomuceno et al., 2021; Yokota et al., 2019; Zaninotto et al., 2020); however, there are very few studies in India which utilize DFLE to examine the sex gap in health. In India, Bora and Saikia (2015), on the basis of self-reported activity limitation with a reference period of 30 days, examined sex differences in health using DFLE and found that females had a higher LE and DLE than males, and the proportion of their life spent in disability was higher than males. Sreerupa et al. (2019), utilizing the indoor and outdoor mobility limitations, found that an increase in ‘LE without mobility limitation’ and a decrease in ‘proportion of life with mobility limitation’ between 1995-96 to 2004 indicates compression of morbidity among older males and older rural dwellers. However, an increase in the ‘LE with mobility limitation’ and an increase in the ‘proportion of life spent with mobility limitation’ in the same period indicates the expansion of morbidity among older females and urban dwellers (Sreerupa et al., 2019). Mishra et al. (2020) used the Census 2011 information on the prevalence of eight disabilities (i.e., seeing, hearing, speech, movement, mental retardation, mental illness, any illness, and any other disability) and found that DFLE was higher among females than males, with significant regional disparities. Thomas et al. (2014) examined LE and DFLE among males and females at age 60 and found a higher LE and DLFE among females than males at the national level and among states. They utilized health indicators such as morbidity, restricted mobility, and being confined to bed for 15 days prior to the survey as indicators of health to compute DLFE. Shabnam and Saikia (2024) used World Health Survey 2003 data to examine the healthy LE (HLE) and morbidity-free LE (MFLE) on the basis of ailments and their spells 15 days prior to the survey and found that the HLE was higher among males than females; however, the MFLE was higher among females than males.

This existing research, however, is limited in several key ways. First, these studies are based on older datasets; for instance, Bora and Saikia (2015) utilized the World Health Organization’s Study on global AGEing and adult health (WHO SAGE) 2007, Sreerupa et al (2019) utilized National Sample Survey (NSS) 52nd (conducted in 1995-96) and NSS 60th (conducted in 2004) round dataset. Second, existing studies are national-level studies, lacking insights into sub-national disparities (Bora & Saikia, 2015; Shabnam & Saikia, 2024; Sreerupa et al., 2019). India is a diverse country where huge inequality exists at the sub-national (state) level. Indian states are heterogeneous in various social and economic development indicators. Evidence suggests that there exists a clear north–south divide in the standard of living at the state level where southern states such as Kerala, Tamil Nadu, Karnataka, and Andhra Pradesh have better standards of living, whereas northern states such as Bihar, Uttar Pradesh, Jharkhand, and Haryana lag behind (Kumar & Rani, 2019; Tyagi, 2023). Apart from economic disparities, there exist inequalities in other demographic characteristics where some states have a younger age-sex structure (i.e., Uttar Pradesh, Bihar, Chhattisgarh), and some are facing population ageing more severely than others (i.e., Kerala) (Dandona et al., 2017). Additionally, infant and child mortality also vary across states. While the national infant mortality rate was 28 (per 1000 live births) in 2020, at the state-level, infant mortality rates ranged as high as 43 in Madhya Pradesh and as low as 6 in Kerala (ORGI, 2022). State-level heterogeneity depicts a clear north–south gradient where northern states such as Uttar Pradesh, Madhya Pradesh, Chhattisgarh, and Odisha have lower levels of child nutrition, female literacy, and wealth and higher levels of infant and child mortality compared to southern states such as Kerala, and Tamil Nadu (Dandona et al., 2020; Singh et al., 2011). Inequalities between states become even more severe by urban–rural residence and social groups (Oxfam India, 2021). Given huge state-level disparities in India, national-level estimates do not suffice to inform policy formulation and implementation. Third, existing studies on DFLE are not explicitly focused on older adults aged 60 and above and did not decompose the sex gap in DFLE in mortality and disability (~health) components (Bora & Saikia, 2015; Mishra et al., 2020).

To fill these important gaps in the existing literature and advance the global discourse on sex gaps in the health of older adults, we aimed to assess the sex (female–male) gap in health (i.e., through LE, DFLE, DLE) and morbidity-mortality paradox among males and females aged 60 years and above in India and its states. Our study assesses disability using questions based on the Katz scale of independence to represent the health component for DLFE estimation with a longer reference period of three months for disability problems. The Katz scale is a comprehensive and widely used disability measure, depicting the chronic impact of non-fatal health and disability (Katz et al., 1963). We estimated DFLE and DLE by combining the most recent available age-specific disability prevalences (ASDP) and age-specific death rates (ASDR) obtained from the Longitudinal Ageing Survey in India (LASI) and the Sample Registration System (SRS), respectively. We first estimated LE at age 60 and above, then decomposed it into DFLE and DLE for males and females to assess sex gap in the health of older adults. Then we decomposed the sex gap in DFLE into mortality and disability components by five-year age groups. Finally, we discuss the implications of sex gaps in monitoring older adults’ health and well-being, which are crucial for achieving healthy ageing, health for all, and the Sustainable Development Goals.

## Methods and materials

### Data source

**Fig. 1:**
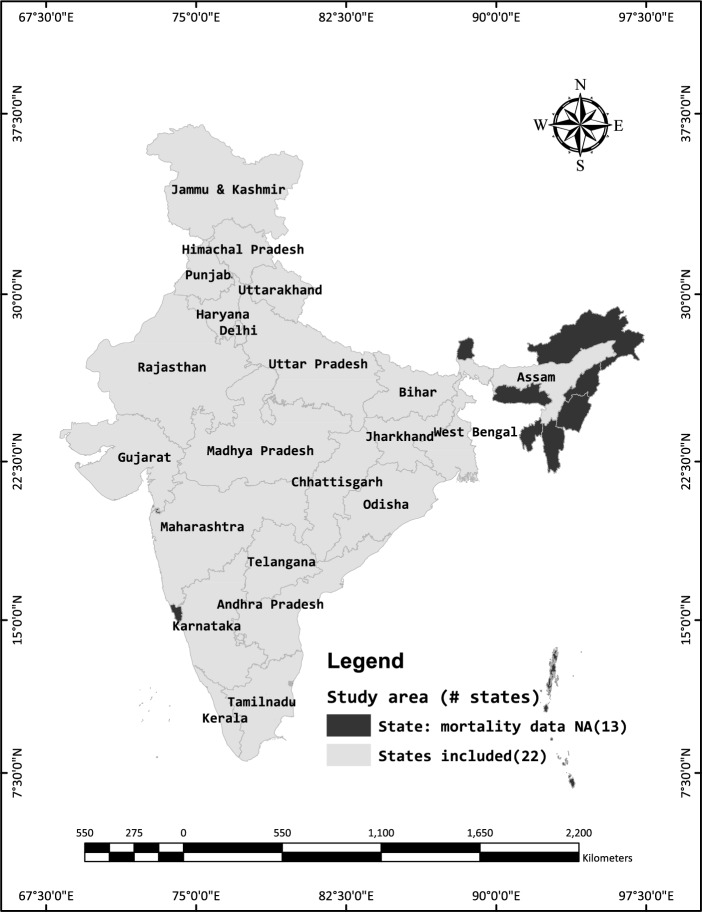
Map showing twenty-two states/union territories included in the study. **Note:** NA = Not Available. ‘Jammu & Kashmir’ and ‘Ladakh’, which are now two separate unions/territories, were part of a single state named Jammu & Kashmir earlier; therefore, total number of states and UTs shown in the map is 35 and not 36 as of present. The names of states are shown only for the states included in this study

We need data on mortality and health (disability) components to estimate health expectancies (i.e., DFLE and DLE). Our mortality data come from the Sample Registration System (SRS) statistical reports (2016–2020). SRS, which has been conducted annually by the Office of Registrar General and Census Commissioner, Government of India, since 1971, provides annual data on population composition, fertility, and mortality indicators collected on the basis of a dual record system. The dual record system of SRS is unique in that the data are collected by two independent functionaries, which are crosschecked on a retrospective half-year basis; unmatched and partially matched information is verified and corrected by revisiting the household (ORGI, 2020). Though SRS collects data across India, it provides estimates only nationally and for states with populations of more than 10 million. In SRS, mortality data at the sub-national level are available for twenty-two states (out of a total of 36 states and union territories [UTs] as of 2024), restraining our analysis at sub-national levels only in those 22 states (Fig. 1). It should be noted that SRS provides the data in the form of reports; raw data are not available in the public domain. Because of the lack of information on sample sizes due to the inaccessibility of raw data, we could not calculate standard errors and confidence intervals of the estimates presented in this paper.

Disability data was obtained from the Longitudinal Ageing Survey (LASI) in India. India’s LASI is similar to Health and Retirement Studies (HRS) conducted in many countries worldwide. It is India’s largest sample survey, providing information on health status and various dimensions of well-being of older adults aged 45 and above, along with their spouses. It is conducted by the International Institute for Population Sciences (IIPS) in collaboration with Harvard University and the University of Southern California (USC). The first wave of LASI was conducted in 2017-18, which provides data on all states and union territories in India (IIPS, 2020). However, as we have mortality data only for 22 states shown in Fig. 1, we obtained disability data for only those states from the LASI. Our sample size for selected twenty-two states is 24,947, but our national sample size is 31,902, which included information from every state of India to make it comparable with SRS’ mortality estimates for India. Detailed sample characteristics are provided in Table S1 in the supplementary file.

### Quality of data obtained from SRS and LASI

The SRS is the most reliable and widely used source of mortality data in India. A number of studies have examined the quality of SRS data in the recent past (Bhat, 2002; Ram et al., 2010, 2021; Saikia et al., 2010; Yadav & Ram, 2019). The coverage of death in SRS is above 90%; 94% for males and 91% for females (Bhat, 2002; Saikia et al., 2010). States such as Maharashtra, Punjab, Andhra Pradesh, Gujarat, Karnataka, Madhya Pradesh, Odisha and Tamil Nadu had almost 100% death coverage during the decade of 2001–2010 but states such as Assam, Bihar Haryana, Himachal Pradesh, Kerala, Rajasthan, Uttar Pradesh, and West Bengal recorded relatively higher undercount of deaths (Yadav & Ram, 2019). Owing to its high quality, it is utilized by Indian central and state governments in various health planning and policy formulations.

LASI is the largest survey on older adults (aged 45 and above) in the world, with an all-India individual response rate of 87.3% which is closer to other health and retirement studies of the world such as 87% in wave 13 (2014) of Health and Retirement Study of USA and 86% in wave 4 (2018) of China Health and Retirement Longitudinal Study (Bloom et al., 2021; Perianayagam et al., 2022a). The individual response rates in LASI varied among states ranged from 77% in Gujarat to 93.9% in Andhra Pradesh (Perianayagam et al., 2022a). LASI is widely recognized for high-quality data on different aspects of the quality of life of older adults, and has been utilized in numerous studies (Chauhan et al., 2022; Malik, 2022; Paul et al., 2023; Perianayagam et al., 2022b; Sharma et al., 2021, 2023).

### Estimation of mortality rate and disability prevalence

We utilized the ASDR as a mortality indicator. As SRS provides annual estimates of ASDR in their report, we calculated the average of the ASDRs of 2016 to 2020 as an estimate of ASDR for the year 2018. The ASDP, obtained from LASI, was used as the indicator of disability in our study. The disability prevalence was measured through the questions based on the Katz scale of independence in the activities of daily living such as dressing, bathing, walking across a room, eating, getting in and out of bed, and toileting which are very fundamental to normal daily self-care. The respondents were asked to answer “yes” if they had any of the given six difficulties which lasted for three months or more, “Do you have any difficulty with dressing, including putting on chappals [sandals], shoes, etc.?”; “Do you have any difficulty with walking across a room?”; “Do you have any difficulty with bathing?”; “Do you have any difficulty with eating?”; “Do you have any difficulty with getting in or out of bed?”; “Do you have any difficulty with using the toilet, including getting up and down?”. Respondents were defined as disabled if they answered “yes” to any of the above questions. This definition has also been used by several recent studies (Halder et al., 2023; Malik, 2022; Sharma et al., 2021).

### Statistical analysis

We first estimated state-wise LE, DFLE, and DLE by sex. Then we calculated sex gaps in estimated LE, DFLE and DLE. The morbidity-mortality paradox was assessed using percentages of LE and ‘additional years of females obtained through mortality advantage’ spent in disability and disability-free states. Finally, the sex gaps in DFLE and DLE were decomposed into mortality and disability components. A detailed account of estimation and calculations is given below:

The LE at age 60 and above was estimated by constructing abridged life tables of five-year age groups comprising 60–64, 65–69, 70–74, 75–79, 80–84, and 85+ . The ASDRs were transformed into probability of dying between ages x and x + n (_n_q_x_) using Greville’s formula given below:

1 $${{}_{\text{n}}}{{\text{q}}_{\text{x}}} = {\text{}}\frac{{{{}_{\text{n}}}{{\text{m}}_{\text{x}}}}}{{\frac{1}{{\text{n}}} + {{}_{\text{n}}}{{\text{m}}_{\text{x}}} \times [\frac{1}{2} + \frac{{\text{n}}}{{12}}({{}_{\text{n}}}{{\text{m}}_{\text{x}}} - \log _{{\text{e}}} {\text{c}})]}}$$

Here x is the starting age of the five-year age group (i.e., 60, 65, 70), n is the age interval (i.e., 5 years), and _n_m_x_ is the age-specific death rate between age x to x + n. The value of log_e_c was taken as 0.095 (Greville, 1943). The estimated probabilities of dying were used to estimate sex-stratified life tables for older adults age 60 years and above.

Sex gaps in health were assessed through the DFLE, a methodological approach suggested by Sullivan (1971) to divide LE into two components, namely DFLE and DLE. In this method, ASDPs are combined with number of person-years lived, calculated from a life table, to estimate number of person-years lived with and without disability. We used the following formula provided by Sullivan (1971), elaborated in detail by Jagger et al. (2007), to estimate the number of person-years lived with and without disability in India and its 22 states:

2 $${{}_{\text{n}}}{\text{L}}_{{\text{x}}}^{{\text{i}}} = {{}_{\text{n}}}{\text{L}}_{\text{x}}\left( {1 - {{}_{\text{n}}}{\uppi_{\text{x}}}} \right){\text{}}$$

3 $${{}_{\text{n}}}{\text{L}}_{\text{x}}^{-\text{i}}={{}_{\text{n}}}{\text{L}}_{\text{x}} \left({{}_{\text{n}}}{{\uppi}_{\text{x}}}\right), $$

where _n_L_x_ is the number of person-years lived between age x to x + n, _n_L_x_^i^ is the number of person-years lived without disability between age x to x + n, _n_L_x_^−i^ is the number of person-years lived with disability between age x to x + n, and _n_π_x_ is the prevalence of disability between age x to x + n, _n_π_x_ was subtracted from 1 to get the proportion of people living without disability or in a healthy state between age x to x + n.

We then calculated the DFLE and DLE using the formula:

4 $$\begin{array}{c}{\text{DFLE}}_{\text{x}}= \frac{\sum_{\text{k}=\text{x}}^{\text{w}}\left({{}_{\text{n}}}{\text{L}}_{\text{k}}^{\text{i}}\right)}{{\text{l}}_{\text{x}}}, \end{array}$$

5 $$\begin{array}{c}{\text{DLE}}_{\text{x}}= \frac{\sum_{\text{k}=\text{x}}^{\text{w}}\left({{}_{\text{n}}}{\text{L}}_{\text{k}}^{\text{-i}}\right)}{{\text{l}}_{\text{x}}}, \end{array}$$

where DFLE_x_ is disability-free LE at age x, DLE_x_ is the LE with disability at age x, w is the starting age of the last open age group, _n_L_k_^i^ is the number of person-years lived without disability (Eq. 2), _n_L_k_^−i^ is the number of person-years lived with disability (Eq. 3) and l_x_ is the number of persons surviving at age x.

We calculated LE, DFLE, and DLE separately for male and female. The sex gap between them was assessed using the following formulas:

6 $$\begin{array}{c}{\Delta \text{LE}}_{\text{x}}= {\text{LE}}_{\text{x}}^{\text{Female}}- {\text{LE}}_{\text{x}}^{\text{Male}}, \end{array}$$

7 $$\begin{array}{c}{\Delta \text{DFLE}}_{\text{x}}={\text{DFLE}}_{\text{x}}^{\text{Female}} - {\text{DFLE}}_{\text{x}}^{\text{Male}}, \end{array}$$

8 $$\begin{array}{c}{\Delta \text{DLE}}_{\text{x}}= {\text{DLE}}_{\text{x}}^{\text{Female}}- {\text{DLE}}_{\text{x}}^{\text{Male}}, \end{array}$$

where _x_ represents the starting age of age groups used in the abridged life table and delta (∆) represents for sex gap. A positive value of ∆LE represents females living longer than males, which is termed as females’ additional years of life obtained through mortality advantage in this entire article.

After estimating LE, DFLE, DLE and their sex gaps, we employed two approaches to assess whether the morbidity-mortality paradox is applicable in India or not. The first approach is based on total LE, whereas the second approach considers the additional years of life females obtained through mortality advantage. For the first approach, we calculated the per cent of total LE at age 60 spent in disability-free (DFLE) and disabled (DLE) states for male and female separately using the following formula:

9 $$\begin{array}{c}{\text{DFLE}}_{60} \left(\text{\%}\right)= \frac{{\text{DFLE}}_{60}}{{\text{LE}}_{60}}\times 100,\end{array}$$

10 $$\begin{array}{c}{\text{DLE}}_{60}\left(\text{\%}\right)= \frac{{\text{DLE}}_{60}}{{\text{LE}}_{60}}\times 100. \end{array}$$

**Fig. 2:**
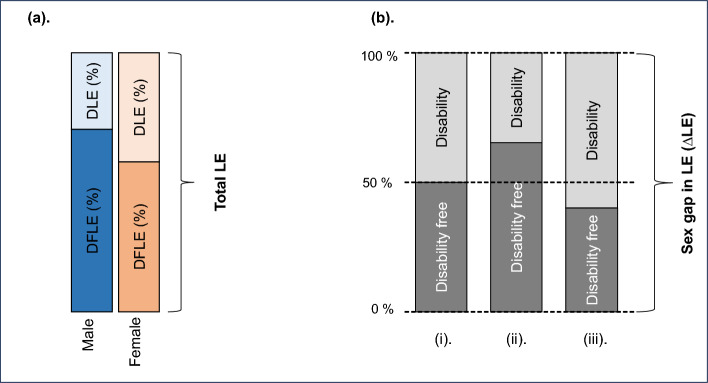
Schematic diagram of the morbidity-mortality paradox

Morbidity-mortality paradox exists when the per cent of total LE spent in the disabled states among females is higher than the per cent of total LE spent in the disabled states among males (Fig. 2a).

For the second approach, we calculated the percentage of additional years of life obtained through mortality advantage spent in disability-free and disabled states. This was assessed through formulas 11 and 12.

11 $${\text{Additional years spent in disability free state}}\,(\%)= \frac{{\Delta \text{DFLE}}_{60}}{{\Delta \text{LE}}_{60}}\times 100,$$

12 $${\text{Additional years spent in disabled state}}\,(\%)= \frac{{\Delta \text{DLE}}_{60}}{\Delta {\text{LE}}_{60}}\times 100. $$

If the percentage of additional years spent in disability and disability-free states are equal (i.e., 50%), then there is a state of equilibrium (Fig. 2b (i)). If the per cent of additional years spent in a disability-free state is more than the per cent spent in disability, then it represents an ideal condition, which should be where most of the mortality advantage obtained is spent in a disability-free or healthy state (Fig. 2b (ii)). But if the percentage of additional years spent in disability is higher than the percentage spent in the disability-free state, then this is a sign of the existence of the morbidity-mortality paradox, which shows that most of females’ mortality advantage is spent in disability (Fig. 2b (iii)).

Finally, we decomposed the sex gap in LE into disability and disability-free states and the sex gap in DFLE and DLE into mortality and disability effects. Decomposition of the sex gap in LE into disability and disability-free components is very simple, and is the sum of the sex gap in DLFE and DLE (Eqs. 13 and 14):

13 $$\begin{array}{c}{\text{LE}}_{\text{x}}^{\text{Female}}- {\text{LE}}_{\text{x}}^{\text{Male}}= \left({\text{DFLE}}_{\text{x}}^{\text{Female}}- {\text{DFLE}}_{\text{x}}^{\text{Male}}\right)+\left({\text{DLE}}_{\text{x}}^{\text{Female}}- {\text{DLE}}_{\text{x}}^{\text{Male}}\right) \end{array}$$

14 $$\begin{array}{c}{\text{LE}}_{\text{x}}^{\text{Female}}- {\text{LE}}_{\text{x}}^{\text{Male}}= \left({\Delta \text{DFLE}}_{\text{x}}\right)+\left({\Delta \text{DLE}}_{\text{x}}\right). \end{array}$$

The decomposition of DLFE and DLE at age x into mortality and disability components was conducted using a step-wise replacement method developed by Andreev et al. (2002). This decomposition method alters one element of a parameter at a time and recalculates the aggregate measure to get the contribution of that element of the parameter in creating a difference in aggregate measure between the groups. In the end, the total contribution of the parameter is obtained simply by summing the contribution of each element of that parameter (Andreev et al., 2002). The mathematical formula of the step-wise replacement decomposition method used in this study to obtain λ_x_ (contribution due to mortality differences at age x) and γ_x_ (contribution due to disability difference at age x) is given in Eqs. 15, 16 and 17 below where i = 1 and 2 shows male and female, respectively:

15 $$\begin{array}{c}{\uplambda }_{\text{x}}=0.25\left({\text{l}}_{\text{x}}^{1}+ {\text{l}}_{\text{x}}^{2}\right)\left({\text{P}}_{\text{x}}^{2}- {\text{P}}_{\text{x}}^{1}\right)\left({\uppi }_{\text{x}}^{1}+{\uppi }_{\text{x}}^{2}\right)+0.5 \left({\text{DFLE}}_{\text{x}+1}^{1}{\text{ l}}_{\text{x}}^{2}+{\text{DFLE}}_{\text{x}+1}^{2} {\text{l}}_{\text{x}}^{1}\right)\left({\text{q}}_{\text{x}}^{1}-{\text{q}}_{\text{x}}^{2}\right), \end{array}$$

16 $$\begin{array}{c}{\upgamma }_{\text{x}}=0.25\left({\text{l}}_{\text{x}}^{1}+ {\text{l}}_{\text{x}}^{2}\right)\left({\text{P}}_{\text{x}}^{1}+ {\text{P}}_{\text{x}}^{2}\right)\left({\uppi }_{\text{x}}^{2}-{\uppi }_{\text{x}}^{1}\right), \end{array}$$

17 $$\begin{array}{c}{\text{DFLE}}_{\text{x}}^{2}- {\text{DFLE}}_{\text{x}}^{1}= \sum_{\text{j}=\text{x}}^{\upomega }{\uplambda }_{\text{x}}+ \sum_{\text{j}=\text{x}}^{\upomega }{\upgamma }_{\text{x}}. \end{array}$$

In the above equations, sub-script x represents the five-year age group, x + 1 represents the next age group, P_x_ denotes the ratio of L_x_^i^ (number of person year lived at age x) to l_x_^i^ (persons surviving at age x), π_x_ is disability prevalence at age x, and $\sum_{j=x}^{\omega }{\lambda }_{x}$ is total contribution of mortality difference or mortality effect and $\sum_{j=x}^{\omega }{\gamma }_{x}$ is total contribution of disability difference or disability effect. The same formula was used to decompose the sex gap in DLE into mortality and disability effects by replacing DLEs with DFLEs of respective sex.

## Results

### Mortality advantage of females

**Fig. 3:**
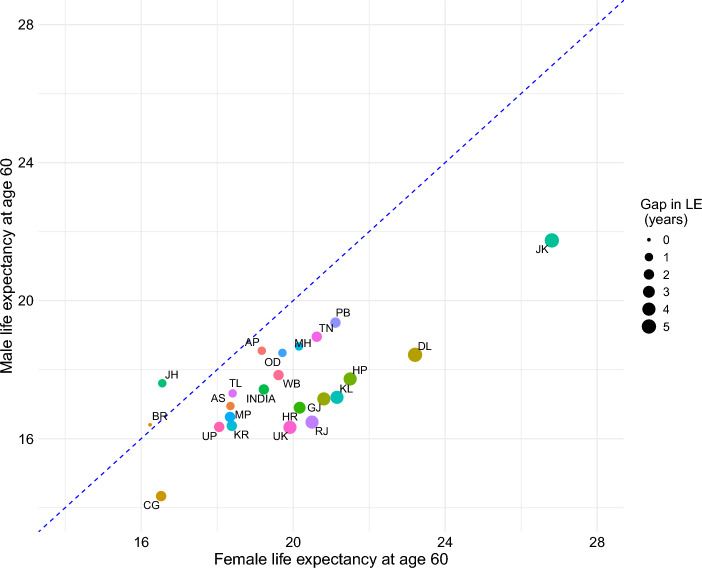
Life expectancy at age 60 and sex gap in life expectancy, India and States, 2018. **Note: **The dashed blue diagonal line is the line of equality for the LE at age 60 for male and female. BR-Bihar, JH-Jharkhand, TL-Telangana, AP-Andhra Pradesh, OD-Odisha, CG-Chhattisgarh, UP-Uttar Pradesh, KR-Karnataka, AS-Assam, PB-Punjab, MH-Maharashtra, TN-Tamil Nadu, HR-Haryana, MP-Madhya Pradesh, UK-Uttarakhand, WB-West Bengal, GJ-Gujarat, KL-Kerala, RJ-Rajasthan, HP-Himachal Pradesh, DL-Delhi, JK-Jammu & Kashmir

At the national level, LE at age 60 (e_60_) for males were 17.4 years and for females were 19.2 years (Fig. 3). The LE for females ranged between 26.8 years in Jammu & Kashmir and 16.2 years in Bihar. The LE for male was smaller, ranging from 21.7 years in Jammu & Kashmir to 14.3 years in Chhattisgarh. Except in Jharkhand and Bihar, LE favoured females in all states (Fig. 3).

### Disability disadvantage

**Fig. 4:**
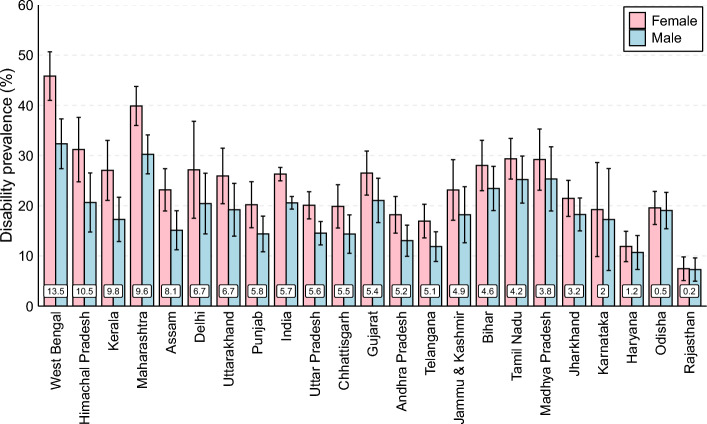
Sex gap in prevalence of disability at age 60 and above, India and States, 2018. **Note:** Data labels displayed on the bars show the sex gap in disability prevalence at ages 60 and above. The error bars show the 95% confidence intervals

The mortality advantage of females is contrasted by disability disadvantage. As shown in Fig. 4, the disability prevalence among females aged 60 and above is higher than males in every state as well as at the national level. The sex gaps in disability were highest in West Bengal, where the proportion of disabled females was 13.5 percentage points higher than for males. This difference was lowest in Rajasthan. The sex gaps in disability were higher than the national average in eight states (West Bengal, Himachal Pradesh, Kerala, Maharashtra, Assam, Delhi, Uttarakhand, and Punjab), and lower than the national average in the states including Uttar Pradesh, Chhattisgarh, Gujarat, and Andhra Pradesh. As females are living longer but also have higher disability rates, assessing mortality and disability separately does not provide a comprehensive picture of health and does not answer the question of whether additional years of life due to mortality advantage among females is spent in full health (disability-free state) or health worse than full health (disabled state).

### Sex gap in LE, DFLE and DLE

Table 1 shows the estimates of LE, DFLE, and DLE at age 60 and above along with the sex gaps, where a positive sign shows advantage for females and a negative sign shows advantage for males. At the national level, the sex gap in LE at age 60 was 1.80 years, with substantial variations among states. Among states, the sex gap in LE was highest in Jammu & Kashmir (5.06 years), followed by Delhi (4.78 years), Rajasthan (4.01 years), Kerala (3.95 years) and Himachal Pradesh (3.77 years).

**Table 1:** LE, DFLE, DLE and their respective sex gaps at age 60, India and States, 2018

State	LE		DFLE		DLE		Sex gap		
	LE (M)	LE (F)	DFLE (M)	DFLE (F)	DLE (M)	DLE (F)	∆LE	∆DFLE	∆DLE
Andhra Pradesh	18.55	19.17	15.57	15.01	2.98	4.17	0.63	−0.56	1.19
Assam	16.95	18.35	14.28	13.09	2.66	5.26	1.40	−1.20	2.60
Bihar	16.40	16.23	12.58	11.38	3.82	4.86	−0.17	−1.20	1.03
Chhattisgarh	14.34	16.52	12.21	12.77	2.13	3.76	2.19	0.55	1.63
Delhi	18.43	23.21	14.36	15.05	4.07	8.15	4.78	0.70	4.08
Gujarat	17.15	20.81	13.31	14.06	3.84	6.75	3.65	0.75	2.91
Haryana	16.90	20.17	15.00	16.95	1.90	3.22	3.27	1.96	1.31
Himachal Pradesh	17.73	21.50	13.95	14.29	3.79	7.21	3.77	0.35	3.42
Jammu & Kashmir	21.75	26.81	15.53	17.94	6.22	8.87	5.06	2.41	2.65
Jharkhand	17.61	16.55	14.10	12.85	3.51	3.70	−1.05	−1.25	0.20
Karnataka	16.37	18.38	13.71	14.75	2.66	3.63	2.01	1.04	0.97
Kerala	17.20	21.15	14.22	14.70	2.98	6.45	3.95	0.48	3.47
Madhya Pradesh	16.63	18.34	12.33	12.90	4.30	5.44	1.71	0.57	1.14
Maharashtra	18.67	20.15	12.69	11.39	5.98	8.77	1.48	−1.31	2.78
Odisha	18.49	19.72	14.30	15.33	4.19	4.39	1.23	1.03	0.20
Punjab	19.36	21.11	16.39	16.33	2.98	4.78	1.75	−0.06	1.81
Rajasthan	16.48	20.50	15.14	18.78	1.34	1.71	4.01	3.64	0.37
Tamil Nadu	18.95	20.62	13.77	13.94	5.18	6.68	1.67	0.16	1.50
Telangana	17.31	18.41	15.07	15.04	2.24	3.37	1.09	−0.03	1.13
Uttar Pradesh	16.34	18.05	13.97	14.10	2.37	3.96	1.71	0.12	1.59
Uttarakhand	16.33	19.91	12.87	13.60	3.46	6.31	3.59	0.73	2.85
West Bengal	17.84	19.61	11.70	10.24	6.15	9.38	1.77	−1.46	3.23
**India**	**17.43**	**19.23**	**13.61**	**13.64**	**3.82**	**5.59**	**1.80**	**0.03**	**1.77**

**Note: **M is used as an abbreviation for males and F for females

The sex gap in DFLE at the national level was 0.03 years, with substantial state-level variations. The highest gap was found in Rajasthan (3.64 years), followed by Jammu & Kashmir (2.41 years), Haryana (1.96 years), and Karnataka (1.04 years), showing that females were living more absolute years in a healthy state than males in these geographies. The lowest gap in DFLE was found in Uttar Pradesh (0.1 years). In contrast, the sex gap in DFLE was negative in West Bengal (−1.46 years), Maharashtra (−1.31 years), Jharkhand (−1.25 years), Assam (−1.20 years), Punjab (−0.06 years), and Telangana (−0.03 years), indicating that males in these geographies live more absolute years in disability-free state than females (Table 1).

The sex gap in DLE was 1.77 years in India, which means that females live 1.77 more years in disability than males at age 60 and above. The sex gap in DLE was highest in Delhi (4.08 years), followed by Kerala (3.47 years), Himachal Pradesh (3.42 years), West Bengal (3.23 years) and Gujarat (2.91 years), whereas it was lowest in Odisha (0.20 years) and Jharkhand (0.20 years).

### Morbidity-mortality paradox

**Fig. 5:**
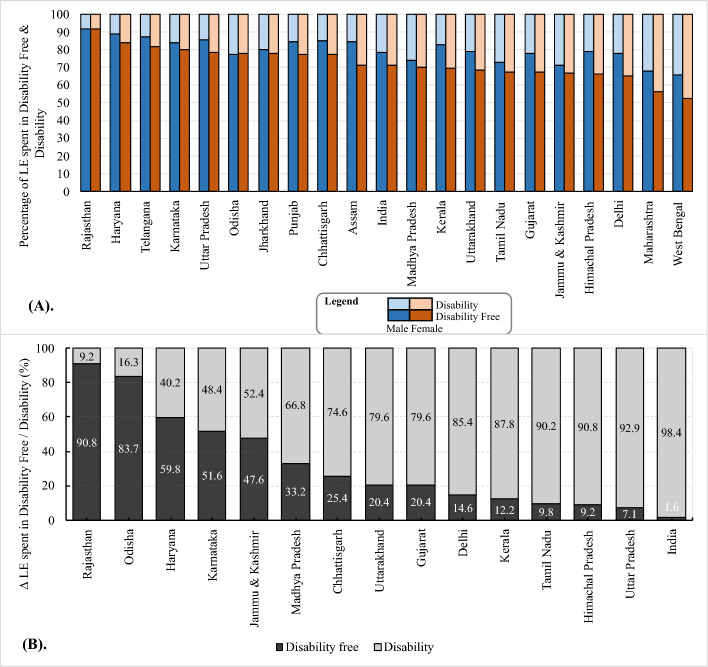
Morbidity-mortality paradox in India and its states: **a** percentage of total LE at age 60 spent in disability-free and disability; **b** percentage of additional years of life (positive ∆LE) at age 60 of females spent in disability-free and disability states, India and States, 2018. **Note:** In Fig. 5(b), we excluded states such as Assam, Maharashtra, Punjab, Telangana, and West Bengal because out of the total mortality advantage of females, the per cent spent in disability states was more than 100% and the percentage spent in disability-free states was negative (less than 0%) in these states. Including these states would have resulted in bars exceeding 100% or falling below 0%, potentially distorting the graph and reducing its readability (see Table S3 in supplementary file)

Due to varying LE at age 60 for females and males, the sex gap in LE, DFLE, and DLE in absolute years hides the true picture of sex gap in health. Hence, it is important to examine the relative contribution (in percentage) of DLFE and DLE to LE and the percentage contribution of ∆DFLE and ∆DLE to ∆LE at age 60 and above by sex. This approach gives a clearer insight into the proportion of total life spent in disability-free and disabled states and the proportion of additional years of life spent in disability-free and disability conditions, respectively. Additionally, examining percentage contribution helps understand whether the morbidity-mortality paradox exists or not. Figure 5a shows that of the total LE at age 60, the proportion of LE spent in disability is higher among females than males in India. In all states except Odisha, the proportion of LE spent in disability for females was higher than for males. The proportion of LE spent in disability was lowest in Rajasthan, where 8.1% of LE for males is spent in disability compared to 8.4% of LE for females spent in disability. It was highest in West Bengal, where males spent 34.4% of their LE in disability compared to females, who spent 47.8% of their LE in disability. These statistics indicate the existence of morbidity-mortality paradox in these states. In Odisha, though very minimal, males spent 22.6% of LE in disability compared to 22.3% among females (Table S2 in supplementary file).

Furthermore, the sex gap in ‘per cent of LE spent in disability’ was highest in West Bengal (13.4 percentage points (pp)) followed by Kerala (12.2 pp), Assam (13 pp), Himachal Pradesh (12.2 pp), and Maharashtra (12.2 pp). It was lowest in Odisha (−0.4 pp), followed by Rajasthan (0.2 pp) and Jharkhand (2.5 pp) (see Table S2 in supplementary file).

To examine the existence of morbidity-mortality paradox using the second approach, we divided ∆DFLE and ∆DLE by ∆LE and multiplied by one hundred; the resulting percentages are shown in Fig. 5b. For some states, of the total mortality advantage of females (positive value of ∆LE), the percentage spent in disability exceeded 100%. For such states, we have shown the values as a multiple of ∆LE (instead of percentages). From second approach as depicted in Fig. 5b, 98.4% of the mortality advantage of females was spent in disability at the national level indicating the existence of morbidity-mortality paradox. The disability disadvantage of females over mortality advantage was highest in Uttar Pradesh; 92.9% of additional years of life were spent with disability. The disability disadvantage of females over mortality advantage was lowest in Rajasthan where only 9.2% of additional years were spent in disability. Except for Rajasthan (9.2%), Odisha (16.3%), Haryana (40.2%) and Karnataka (48.4%), in all other assessed states, more than 50% of the additional years of the life of females were spent in disability. The disability disadvantage of females over mortality advantage was more than 80% in Delhi (85.4%), Kerala (87.8%), Tamil Nadu (90.2%), Himachal Pradesh (90.8%), and Uttar Pradesh (92.9%). On the other hand, Maharashtra, Assam, West Bengal, Punjab, and Telangana were the states where the time spent in disability by females is higher than their total mortality advantage by 1.89, 1.85, 1.83, 1.04, and 1.03 times, respectively (Table S3 in supplementary file).

In sum, a higher proportion of females’ lives, both total LE and additional years of life (∆LE) due to mortality advantage, were spent in a disabled state. This shows the existence of a morbidity-mortality paradox among older adults in India and a majority of its states.

### Decomposition of the sex gap in DLE and DFLE

Decomposition of the sex gap in DFLE and DLE into mortality and disability effects can further enhance our understanding of which factor plays a crucial role and what ages shape that gap. Table 2 shows the results of the step-wise decomposition used to decompose the sex gap in DLE and DFLE into mortality and disability effects. DLE’s decomposition suggests that in most of the states, the mortality and disability effects contributed to increasing the sex gap in DLE; females were living longer years with disability because they were surviving more (mortality advantage) and had higher levels of disability (disability disadvantage). In Assam, Maharashtra, West Bengal, Punjab, Jharkhand, Kerala, Himachal Pradesh, Uttar Pradesh, Telangana, Gujarat, Tamil Nadu, Uttarakhand, and Chhattisgarh, the disability effect dominated the mortality effect. On the other hand, the mortality effect was higher than the disability effect in Jammu & Kashmir, Delhi, Rajasthan, Odisha, Haryana, Karnataka, and Madhya Pradesh (Table 2).

**Table 2:** Decomposition of the sex gap in LE, DFLE, and DLE at age 60, India and States, 2018

State	LE			DFLE			DLE		
	Sex gap	DFLE	DLE	Sex gap	ME	DE	Sex gap	ME	DE
Andhra Pradesh	0.63	−0.56	1.19	−0.56	0.48	−1.05	1.19	0.14	1.05
Assam	1.40	−1.20	2.60	−1.20	0.95	−2.15	2.60	0.45	2.15
Bihar	−0.17	−1.20	1.03	−1.20	−0.12	−1.08	1.03	−0.05	1.08
Chhattisgarh	2.19	0.55	1.63	0.55	1.49	−0.93	1.63	0.70	0.93
Delhi	4.78	0.70	4.08	0.70	2.09	−1.40	4.08	2.68	1.40
Gujarat	3.65	0.75	2.91	0.75	2.50	−1.76	2.91	1.15	1.76
Haryana	3.27	1.96	1.31	1.96	2.55	−0.59	1.31	0.72	0.59
Himachal Pradesh	3.77	0.35	3.42	0.35	2.40	−2.05	3.42	1.37	2.05
Jammu & Kashmir	5.06	2.41	2.65	2.41	2.33	0.08	2.65	2.73	−0.08
Jharkhand	−1.05	−1.25	0.20	−1.25	−0.71	−0.55	0.20	−0.35	0.55
Karnataka	2.01	1.04	0.97	1.04	1.51	−0.47	0.97	0.50	0.47
Kerala	3.95	0.48	3.47	0.48	2.60	−2.11	3.47	1.36	2.11
Madhya Pradesh	1.71	0.57	1.14	0.57	1.13	−0.56	1.14	0.58	0.56
Maharashtra	1.48	−1.31	2.78	−1.31	0.79	−2.10	2.78	0.68	2.10
Odisha	1.23	1.03	0.20	1.03	0.82	0.21	0.20	0.41	−0.21
Punjab	1.75	−0.06	1.81	−0.06	1.35	−1.41	1.81	0.40	1.41
Rajasthan	4.01	3.64	0.37	3.64	3.52	0.13	0.37	0.50	−0.13
Tamil Nadu	1.67	0.16	1.50	0.16	1.07	−0.90	1.50	0.60	0.90
Telangana	1.09	−0.03	1.13	−0.03	0.79	−0.83	1.13	0.30	0.83
Uttar Pradesh	1.71	0.12	1.59	0.12	1.22	−1.09	1.59	0.50	1.09
Uttarakhand	3.59	0.73	2.85	0.73	2.33	−1.60	2.85	1.25	1.60
West Bengal	1.77	−1.46	3.23	−1.46	0.86	−2.32	3.23	0.91	2.32
**India**	**1.80**	**0.03**	**1.77**	**0.03**	**1.17**	−**1.14**	**1.77**	**0.63**	**1.14**

**Note: **We first decomposed the sex gap in LE into DFLE and DLE. Thereafter, we decomposed the sex gap in DFLE and DLE into mortality effect (ME) and disability effect (DE)

A higher sex gap in DFLE was found in the states where females’ mortality advantage was very high and surpassed the disability disadvantage, for example, in Rajasthan, Jammu & Kashmir, Haryana, Karnataka and Odisha. On the other hand, at national level, a high prevalence of disability among females (−1.14 years) and equivalent mortality advantage (1.17 years) in combination resulted in males and females having nearly the same number of healthy years at age 60 (sex gap in DFLE = 0.03). The same condition prevailed in Punjab, Telangana, Uttar Pradesh, and Tamil Nadu, where sex gap in DFLE ranged between −0.06 to 0.16, showing almost equal number of healthy years for males and females at age 60.

**Fig. 6:**
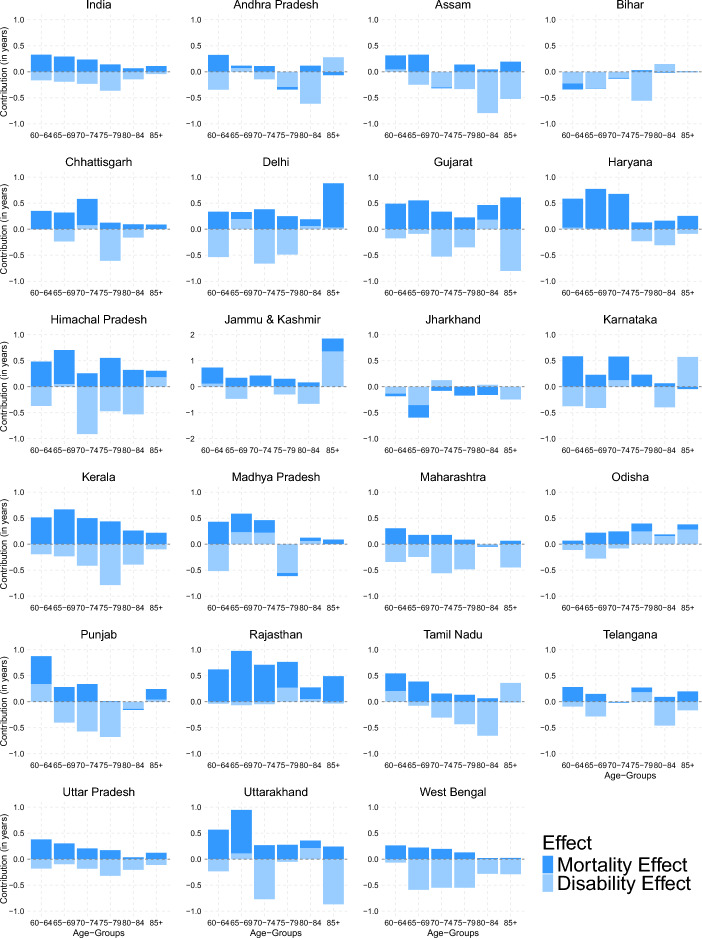
Decomposition of sex gap in DFLE into mortality and disability component by age, India and States, 2018

An age-group-wise decomposition of sex gap in DLFE can give a better understanding of the contribution of mortality and disability effects by age in creating overall female–male differences in healthy years. Such a decomposition may be useful to guide policy formulation and draw the need for specific intervention. Figure 6 depicts the age decomposition of the sex gap in DFLE by five-year age group.

The contribution of mortality to the sex gap in DFLE is higher in most age groups and most states. Mortality’s contribution to the DFLE gap decreases with an increase in age, in most of the states. For example, the contribution of mortality decreases by 50% between age-groups 60–64 and 80–84 in India and Kerala. In contrast, no clear age pattern was found for the contribution of disability effect in DFLE. However, the positive disability effect in age-group 65–69 in Delhi, 80–84 in Gujarat, and at age 85+ in Karnataka shows that the disability prevalence was higher among males than females in these age-groups. On the other hand, the negative mortality effect in all age-groups in Jharkhand, in age-group 75–79 in Madhya Pradesh and at age 85+ in Karnataka shows that the mortality was higher among females than males at these ages (Fig. 6).

## Discussion

Our analysis, using the DFLE approach, shows that females live longer than males in both disabled and disability-free states because of their survival advantage. However, the proportion of both the total LE and the additional years of life due to mortality advantage spent in disability was higher among females than males at the national level and in a majority of states. Of the total LE, the proportion of life spent in disability was higher among females than males in all assessed states except Odisha. Of the additional years of life (mortality advantage) of females, the proportion spent in disability (disability disadvantage) was more than 50% in India and its states, except for Rajasthan, Odisha, Haryana, and Karnataka. These statistics indicate that the mortality-morbidity paradox is applicable in India and all states except Odisha if total life expectancy is considered, whereas it is applicable in India and all states except Rajasthan, Odisha, Haryana, and Karnataka if only additional years due to mortality advantage is considered. Our finding that, despite females living longer than males, female spent more time of their life in disability is consistent with studies conducted in other parts of the world (Andrade et al., 2011; Kulminski et al., 2008; Nepomuceno et al., 2021; Oksuzyan et al., 2018; Yokota et al., 2019) as well as in India (Bora & Saikia, 2015; Mishra et al., 2020; Sreerupa et al., 2019; Thomas et al., 2014).

We found that both DFLE and DLE in absolute years were higher among females than males. The DFLE was higher because females lived longer than males despite having higher disability rates. A higher positive sex gap in DFLE means females’ disability disadvantage is not able to rule out their mortality advantage resulting into females having more absolute disability-free years than males. For example, in Rajasthan, Jammu & Kashmir, Haryana, Karnataka and Odisha a higher mortality effect combined with lesser disability effect resulted in higher positive sex gap in DFLE indicating an advantage for females. However, at the national level, close to equal levels of mortality and disability effect resulted in nearly zero DFLE gap. In Punjab, Telangana, Uttar Pradesh and Tamil Nadu, the DFLE gap was minimal, ranging from −0.06 to 0.16. On the other hand, DLE was higher among females than males due to the combined effect of lesser mortality and higher disability rates among females (Nepomuceno et al., 2021). Additionally, we found that the relative contribution of DLE to LE was higher among females than males, indicating a relatively higher proportion of their life spent in disability than males. The mortality advantage turned into disability disadvantage in every assessed state except Rajasthan, Odisha, Haryana, and Karnataka.

We found that in India overall, and states such as Maharashtra, Assam, West Bengal, Punjab, Telangana, Uttar Pradesh, Himachal Pradesh, Tamil Nadu, Kerala, Delhi, Gujarat, Uttarakhand, Chhattisgarh, Madhya Pradesh, and Jammu & Kashmir, though females lived longer, they spent more time in worse health. Several studies have tried to explain this phenomenon and have proposed biological and social explanations. As per biological explanations, females live longer than males, plausibly due to the presence of xx-chromosome that provide two cell lines that ultimately increases their longevity (Libert et al., 2010; Oksuzyan et al., 2008, 2014). In contrast, the reduction in oestrogen levels post-menopause increases females’ susceptibility to chronic heart disease and several comorbidities, leading them to spend more time in poor health, which becomes more pronounced at older ages (Libert et al., 2010; Oksuzyan et al., 2008, 2014). Conversely, because testosterone causes immunosuppression, the likelihood of producing antibodies is lower among males, leading to their higher mortality (Libert et al., 2010; Oksuzyan et al., 2008, 2014). Patwardhan et al. (2024) found that males tend to have mortality-driven health conditions such as ischaemic heart disease, road injuries, lung cancer and chronic kidney disease, which plausibly result in the early death of males. In contrast, females tend to have a higher burden of morbidity-driven health conditions such as low back pain, headache, and musculoskeletal disorders (Patwardhan et al., 2024). Several Indian studies have reported that older females tend to have a significantly higher risk of bone disease (Anand et al., 2023), knee osteoarthritis (Thati, 2021), high blood pressure (Negi et al., 2022), and a higher level of hypertension after age 50 (Mohanty et al., 2022) than males, which could be attributed to biological changes among them post-menopause.

Gender roles play into these social explanations, with females tending to spend more time inside engaging in household chores, resulting in higher exposure to indoor air pollution (IAP), and increasing their risk for non-fatal chronic, functional, and cognitive illnesses (Bird & Rieker, 2008; Gorman et al., 2006; Oksuzyan et al., 2008; Patwardhan et al., 2024). Several studies based in India have found a negative association between IAP and health status, which is even more pronounced in the elderly population (Rani et al., 2023; Saha et al., 2024). Maharana et al. (2018) found that females exposed to IAP reported symptoms like suffocation, eye irritation, and dry cough compared to unexposed individuals. Studies by Dakua et al. (2022), Rani et al. (2023), and Saha et al. (2024) also suggest that females exposed to IAP have poorer cognitive health than males. Additionally, Lekha et al. (2024) found a higher risk of angina among people living in households using solid fuel for cooking, which disproportionately impacts females due to their role in cooking and proximity to IAP sources within the household. Halder et al. (2024) further strengthened this link by demonstrating that females exposed to IAP have poorer self-rated health compared to males. These findings collectively highlight IAP as a significant risk factor for non-fatal chronic, functional, and cognitive diseases. This association makes females more vulnerable due to existing gender roles that often lead to females’ greater exposure to IAP in the household setting. A very high level of air pollution and associated morbidity levels in Uttar Pradesh, Maharashtra, and West Bengal support our findings (Pandey et al., 2021).

Plausible social explanations suggest that males may be more likely than females to engage in risky behaviours like drinking alcohol, smoking cigarettes, frequent use of psychoactive substances, and driving less safely (Bird & Rieker, 2008; Griswold et al., 2018; Oksuzyan et al., 2008, 2014). These behaviours raise the risk of heart disease, lung cancer, liver cirrhosis, chronic obstructive pulmonary diseases, and accidental deaths (Bird & Rieker, 2008; Griswold et al., 2018; Oksuzyan et al., 2008, 2014). A higher age-standardized suicide death rate (Dandona et al., 2018) and substantially higher premature mortality due to cardiovascular disease (Kundu et al., 2023) among males than females demonstrate health consequences that may be related to riskier behaviours.

Gendered norms related to disclosure of illness may also partially explain this paradox. Males are often less willing to disclose illnesses than females, due to social stigma, as noted in a recent study by Shabnam and Saikia (2023). Nathanson (1975) and Verbrugge (1982) found that males were traditionally more stigmatized by the idea of illness than females. They also found that even psychologists and psychiatrists perceive mental health differently for genders, where males suffering from mental illness are often discouraged, while females suffering from the same illnesses are more often supported and listened to, resulting in females being more willing than males to disclose any health deviations from adult norms because they experience less stigma for doing so (Nathanson, 1975; Verbrugge, 1982). Other plausible reasons for females spending more time in poor health than males despite living longer include a higher prevalence of both physical and mental illnesses among females due to hormonal changes post-menopause (Bambra et al., 2021; Bird & Rieker, 2008; Nathanson, 1975; Rieker & Bird, 2005).

State-level variations in the healthy life years may be attributable to variations in the epidemiological transition level (ETL) and respective burden of diseases. Our finding that the proportion of LE spent in disability was higher for both sexes in states such as West Bengal, Maharashtra, Delhi, Himachal, and Jammu & Kashmir is plausibly due to their higher-middle epidemiological transition level. A relatively higher ETL in these states means a higher disease burden due to noncommunicable diseases, resulting in more years of life lost due to disability, indicating females and males spend more proportion of their lives in a disabled state. On the other hand, Odisha is a state where the morbidity-mortality paradox was not relevant, neither on total LE nor on additional years of life, which is plausibly due to its low epidemiological transition level and resultant lower levels of years of life lost due to disability. States such as Punjab, Himachal Pradesh, Tamil Nadu, and Kerala have most of females’ mortality advantage spent in disability; interestingly, these are the states with the highest levels of epidemiological transition in India (Dandona et al., 2017). A process working behind epidemiological transition and a lesser proportion of DFLE could be that with an increase in epidemiological transition, the prevalence of noncommunicable disease increases, and a higher NCD burden with longer duration results in higher levels of disability resulting in a higher proportion of LE lived with disability (Dandona et al., 2017; Yadav & Arokiasamy, 2014).

In Rajasthan, Odisha, Haryana, and Karnataka, the morbidity-mortality paradox was not applicable. A plausible reason for this could be lower levels of diabetes and neurologic or psychiatric disorders among females than males in Rajasthan (diabetes: 6.8% among females versus 11.9% among males; neurologic or psychiatric disorders: 1.0% versus 2.2%) (IIPS, 2020). A similar pattern was also seen in Haryana and Odisha, where females have lower levels of diabetes and neurologic or psychiatric disorders than males. Lower prevalence of these NCDs might have resulted in a larger proportion of females’ mortality advantage spent in disability-free state in these states.

In West Bengal, proportion of total LE spent in disability and the proportion of mortality advantage spent in disability were higher among older females, resulting in the existence of a morbidity-mortality paradox. A possible reason for this existing condition in West Bengal may be a higher level of self-reported diagnosed chronic conditions among females compared to males. For instance, the prevalence of neurologic/psychiatric disease was 10.7% among females (aged 60 and above) in West Bengal compared to 7.1% in males. Similarly, obesity was 3.4% among females compared to 0.4% among males, and chronic bone disease was 37.6% among females compared to 27.9% among males in West Bengal (IIPS, 2020). Our finding that a higher proportion of LE and a higher proportion of additional years of females spent in disability in Maharashtra could be attributed to a higher prevalence of chronic disease among older females compared to older males. In Maharashtra, compared to males, females have higher levels of chronic bone disease (21.9% among males versus 30.4% among females), higher levels of hypertension (34.3% versus 39.5%) and higher levels of obesity (4.5% versus 8.3%) (IIPS, 2020).

## Robustness check and limitations

We checked our estimates’ reliability by comparing our calculated LE at age 60 with the SRS’s LE for India and its states. Our estimates of LE for males either matched with SRS or was very close at the national level and for a majority of states included in the analysis. Our estimated LE for males differed by more than one year in the states of Delhi (−1.4 years) and Jammu & Kashmir (1.4 years). For females, our calculated LE differed by more than one year from SRS for the states of Himachal Pradesh (−1.6 years) and Jammu & Kashmir (3.9 years). The difference in LE between the two estimates could be due to the start point of the two sets of life tables. The SRS used all the ASDRs, starting from age 0 and ending at age 85+ , for constructing the life tables. In comparison, we used ASDRs starting from age 60–64 to 85+ for constructing the life tables. While our estimates of LE for both male and female are robust enough for India and a majority of states, caution must be exercised when interpreting our results for Jammu & Kashmir, Himachal Pradesh and Delhi.

Only the first wave of LASI has been conducted till now, limiting our capacity to do a trend analysis. Our measure of disability is self-reported, which may be influenced by sex differences in illness-reporting behaviour. However, because of the high individual-level response rates (87.3% at the national level) and the use of standard questions to measure disability, we believe that our self-reported disability measure is reliable. In addition to this, the sample sizes were smaller for the oldest age groups in some states (12 males and 14 females in Uttarakhand and Delhi, respectively, at age 80–84 and 4 females and 5 males in Delhi and Uttarakhand, respectively, at age 85+), which may have compromised disability prevalence rates. Unreliable disability prevalence rates may bias estimates of DFLE and DLE for oldest-old age groups, such as 80–84 and 85+ . We have attempted to mitigate this concern by focusing our analysis of LE, DFLE, and DLE at age 60, where there were larger sample sizes for males and females in all assessed states. Lack of similar studies in the Indian context, with no studies at the state level, limited our ability to externally validate our findings. This underscores the need for more national and state-level studies to explain the ongoing mortality-morbidity paradox in India.

## Conclusion

We assessed the sex gap in health among older adults in India and its states through DFLE. Our study gives a comprehensive snapshot of the health of older adults in India and its states by combining the mortality and morbidity indicators. We found that in India at the national level, and in all states except Rajasthan, Odisha, Haryana, and Karnataka, the mortality advantage of females was spent in disability, showing the existence of a morbidity-mortality paradox. Our finding suggests two policy implications. First, there is an urgent need in India and many of its states to reduce the sex gap in DFLE and DLE, with a particular focus on reducing disability among females. Second, along with addressing the sex gap, there is a need to draw state-specific interventions. For example, Maharashtra, Assam, West Bengal, Punjab, and Telangana require particular attention to reduce disability rates among females, as the proportion of time spent in disabled states in these states is higher than their total mortality advantage (positive ∆LE at age 60).

On the other hand, different interventions are needed in the states of Delhi, Kerala, Tamil Nadu, Himachal Pradesh, and Uttar Pradesh, where more than 80% of females’ mortality advantage is spent on disability. Our findings can potentially guide policymakers to make policies and draw interventions to reduce sex and regional disparities in DLFE and DLE, which will help India “achieve gender equality and empower all women” (SDG-5), “reduce inequality within the country” (SDG-10), and ultimately “ensure a healthy life and promote well-being for all” (SDG-3). Our findings establish that focusing exclusively on LE as an indicator of health does not suffice; examining the DLFE and DLE offers a more nuanced and comprehensive understanding of sex gap in the health status of older adults in India. Our study also adds to the ongoing global debate on the morbidity-mortality paradox by providing a detailed profile of sex gap in healthy LE in India and its states.

## Supplementary Information

Additional file 1.

## Data Availability

We utilized secondary datasets obtained from two sources. The dataset on mortality is available online and can be obtained for free from the website of the Office of the Registrar General & Census Commissioner, India (ORGI) at https://censusindia.gov.in/census.website/data/SRSSTAT. The data on disability used in this study can be obtained for free by submitting an online request to the International Institute for Population Sciences (IIPS), Mumbai repository at https://iipsindia.ac.in/sites/default/files/LASI_DataRequestForm_0.pdf or it can be downloaded by creating a free account on the official website of the International Institute for Population Science (Link: https://www.iipsdata.ac.in/).

## List of Abbreviations

ASDR: Age-Specific Death Rate
HLE: Healthy Life Expectancy
LE: Life Expectancy
DFLE: Disability-Free Life Expectancy
DLE: Life-Expectancy with Disability
∆LE: Sex gap in life expectancy (obtained by subtracting LE for males from LE for females)
∆DFLE: Sex gap in disability-free life expectancy (obtained by subtracting DFLE for males from DFLE for females)
∆DLE: Sex gap in life expectancy with disability (obtained by subtracting DLE for males from DLE for females)
ME: Mortality Effect
DE: Disability Effect
IAP: Indoor Air Pollution
LASI: Longitudinal Ageing Survey in India
SRS: Sample Registration System
ETL: Epidemiological Transition Level
NCD: Non-Communicable Disease
MFLE: Morbidity-Free Life Expectancy
