# Supplementary material for: Sex disparities in health of older adults in India: assessing the morbidity-mortality paradox through disability-free life expectancy
Source: Genus. 2025 May 13;81(1):11. doi: 10.1186/s41118-025-00247-2 (PMC12075278; doi:10.1186/s41118-025-00247-2)
Supplement: Supplementary file 1 — Additional file 1. [file 41118_2025_247_MOESM1_ESM.docx]

**SUPPLEMENTARY FILE**

**Table S1: Sample characteristics obtained from LASI to estimate disability rates across India in 2018, nationally and within states**

| **State** | **Total (N)** | **Male** | | | **Female** | | |
| --- | --- | --- | --- | --- | --- | --- | --- |
|  |  | **%** | **Mean age** | **SD of age** | **%** | **Mean age** | **SD of age** |
|  |  |  |  |  |  |  |  |
| Andhra Pradesh | 1105 | 49.9 | 68.1 | 6.5 | 50.1 | 68 | 7.9 |
| Assam | 816 | 47.5 | 69.1 | 7.4 | 52.5 | 68.4 | 7 |
| Bihar | 1808 | 51.2 | 69 | 7.2 | 48.8 | 68 | 7.5 |
| Chhattisgarh | 780 | 50.3 | 67.7 | 6.6 | 49.7 | 67.8 | 7.2 |
| Delhi | 495 | 50.7 | 68.4 | 7.3 | 49.3 | 67.5 | 6.7 |
| Gujarat | 991 | 45.8 | 68.1 | 6.4 | 54.2 | 67.6 | 6.8 |
| Haryana | 848 | 42.8 | 69.1 | 7.5 | 57.2 | 68.2 | 7.1 |
| Himachal Pradesh | 621 | 49 | 69.7 | 7.8 | 51 | 69.2 | 7.6 |
| Jammu & Kashmir | 731 | 52.3 | 69.9 | 7.9 | 47.7 | 69.4 | 7.9 |
| Jharkhand | 1168 | 50.1 | 68.5 | 7.5 | 49.9 | 68.2 | 7.7 |
| Karnataka | 1004 | 47.7 | 68.7 | 6.6 | 52.3 | 69 | 7.6 |
| Kerala | 1209 | 44.5 | 69.9 | 7.5 | 55.5 | 69.8 | 8 |
| Madhya Pradesh | 1313 | 49 | 69 | 7.8 | 51 | 69.6 | 8.5 |
| Maharashtra | 1790 | 46.3 | 69.1 | 6.9 | 53.7 | 68.6 | 7.3 |
| Odisha | 1237 | 48.6 | 69 | 7.4 | 51.4 | 69 | 7.4 |
| Punjab | 1004 | 49.9 | 69.6 | 7.4 | 50.1 | 68.4 | 7.2 |
| Rajasthan | 1078 | 46.8 | 68.7 | 7.3 | 53.2 | 69.4 | 8.2 |
| Tamil Nadu | 1534 | 44.7 | 69.2 | 7 | 55.3 | 68.9 | 7.2 |
| Telangana | 1061 | 47.3 | 69.5 | 7.2 | 52.7 | 68.9 | 8.1 |
| Uttar Pradesh | 2169 | 51.6 | 69.1 | 7.6 | 48.4 | 68.7 | 8 |
| Uttarakhand | 641 | 47.3 | 67.9 | 6.4 | 52.7 | 67.3 | 6.5 |
| West Bengal | 1544 | 48.7 | 68.9 | 7.7 | 51.3 | 69.8 | 8.4 |
| **India** | **31902** | **48.1** | **69** | **7.4** | **51.9** | **68.8** | **7.7** |

**Table S2: Proportion of disability-free life expectancy (DFLE) and disability life expectancy (DLE) at age 60 by sex across India in 2018, nationally and within states**

| **State** | **Male** | | **Female** | | **Morbidity-mortality**  **paradox** | **Sex gap in DLE (%)** |
| --- | --- | --- | --- | --- | --- | --- |
|  | **DFLE (%)** | **DLE (%)** | **DFLE (%)** | **DLE (%)** |  |  |
| Andhra Pradesh | 83.9 | 16.1 | 78.3 | 21.7 | Yes | 5.7 |
| Assam | 84.3 | 15.7 | 71.3 | 28.7 | Yes | 13.0 |
| Bihar | 76.7 | 23.3 | 70.1 | 29.9 | Yes | 6.6 |
| Chhattisgarh | 85.2 | 14.8 | 77.3 | 22.7 | Yes | 7.9 |
| Delhi | 77.9 | 22.1 | 64.9 | 35.1 | Yes | 13.0 |
| Gujarat | 77.6 | 22.4 | 67.6 | 32.4 | Yes | 10.0 |
| Haryana | 88.7 | 11.3 | 84.1 | 15.9 | Yes | 4.7 |
| Himachal Pradesh | 78.6 | 21.4 | 66.5 | 33.5 | Yes | 12.2 |
| Jammu & Kashmir | 71.4 | 28.6 | 66.9 | 33.1 | Yes | 4.5 |
| Jharkhand | 80.1 | 19.9 | 77.6 | 22.4 | Yes | 2.5 |
| Karnataka | 83.7 | 16.3 | 80.2 | 19.8 | Yes | 3.5 |
| Kerala | 82.7 | 17.3 | 69.5 | 30.5 | Yes | 13.2 |
| Madhya Pradesh | 74.2 | 25.8 | 70.4 | 29.6 | Yes | 3.8 |
| Maharashtra | 68.0 | 32.0 | 56.5 | 43.5 | Yes | 11.5 |
| Odisha | 77.4 | 22.6 | 77.7 | 22.3 | No | -0.4 |
| Punjab | 84.6 | 15.4 | 77.3 | 22.7 | Yes | 7.3 |
| Rajasthan | 91.9 | 8.1 | 91.6 | 8.4 | Yes | 0.2 |
| Tamil Nadu | 72.7 | 27.3 | 67.6 | 32.4 | Yes | 5.1 |
| Telangana | 87.1 | 12.9 | 81.7 | 18.3 | Yes | 5.4 |
| Uttar Pradesh | 85.5 | 14.5 | 78.1 | 21.9 | Yes | 7.4 |
| Uttarakhand | 78.8 | 21.2 | 68.3 | 31.7 | Yes | 10.5 |
| West Bengal | 65.6 | 34.4 | 52.2 | 47.8 | Yes | 13.4 |
| **India** | **78.1** | **21.9** | **70.9** | **29.1** | **Yes** | **7.2** |

**Table S3: Percentage of additional years of life of females (mortality advantage) spent in disability and disability-free states across India in 2018, nationally and within states**

| **State** | **∆LE** | **∆DFLE** | **∆DLE** | **Disability free (%)**  $\left[ \frac{\boldsymbol{\Delta}\mathbf{DFLE}}{\boldsymbol{\Delta}\mathbf{LE}}\boldsymbol{\times}\boldsymbol{100} \right]$ | **With disability (%)**  $\left[ \frac{\boldsymbol{\Delta}\mathbf{DLE}}{\boldsymbol{\Delta}\mathbf{LE}}\boldsymbol{\times}\boldsymbol{100} \right]$ | **Morbidity-**  **mortality**  **paradox** |
| --- | --- | --- | --- | --- | --- | --- |
| *States with female mortality advantage* | | | | | | |
| Andhra Pradesh | 0.63 | -0.56 | 1.19 | -90.31 | 190.31 | Yes |
| Assam | 1.40 | -1.20 | 2.60 | -85.45 | 185.45 | Yes |
| Chhattisgarh | 2.19 | 0.55 | 1.63 | 25.38 | 74.62 | Yes |
| Delhi | 4.78 | 0.70 | 4.08 | 14.55 | 85.45 | Yes |
| Gujarat | 3.65 | 0.75 | 2.91 | 20.42 | 79.58 | Yes |
| Haryana | 3.27 | 1.96 | 1.31 | 59.85 | 40.15 | No |
| Himachal Pradesh | 3.77 | 0.35 | 3.42 | 9.18 | 90.82 | Yes |
| Jammu & Kashmir | 5.06 | 2.41 | 2.65 | 47.64 | 52.36 | Yes |
| Karnataka | 2.01 | 1.04 | 0.97 | 51.65 | 48.35 | No |
| Kerala | 3.95 | 0.48 | 3.47 | 12.23 | 87.77 | Yes |
| Madhya Pradesh | 1.71 | 0.57 | 1.14 | 33.18 | 66.82 | Yes |
| Maharashtra | 1.48 | -1.31 | 2.78 | -88.50 | 188.50 | Yes |
| Odisha | 1.23 | 1.03 | 0.20 | 83.69 | 16.31 | No |
| Punjab | 1.75 | -0.06 | 1.81 | -3.52 | 103.52 | Yes |
| Rajasthan | 4.01 | 3.64 | 0.37 | 90.78 | 9.22 | No |
| Tamil Nadu | 1.67 | 0.16 | 1.50 | 9.84 | 90.16 | Yes |
| Telangana | 1.09 | -0.03 | 1.13 | -3.16 | 103.16 | Yes |
| Uttar Pradesh | 1.71 | 0.12 | 1.59 | 7.08 | 92.92 | Yes |
| Uttarakhand | 3.59 | 0.73 | 2.85 | 20.43 | 79.57 | Yes |
| West Bengal | 1.77 | -1.46 | 3.23 | -82.62 | 182.62 | Yes |
| **India** | **1.80** | **0.03** | **1.77** | **1.56** | **98.44** | Yes |
| *States with female mortality disadvantage* | | | | | | |
| Bihar | -0.17 | -1.20 | 1.03 | Not applicable as sex gap in life expectancy (∆LE)  is not in favour of females. | | |
| Jharkhand | -1.05 | -1.25 | 0.20 |  |  |  |

Note: LE=life expectancy; DFLE=disability-free life expectancy; DLE=disability life expectancy
